# Pain and Other Neurological Symptoms Are Present at 3 Months After Hospitalization in COVID-19 Patients

**DOI:** 10.3389/fpain.2021.737961

**Published:** 2021-11-16

**Authors:** Jude P. J. Savarraj, Angela B. Burkett, Sarah N. Hinds, Atzhiry S. Paz, Andres Assing, Shivanki Juneja, Gabriela D. Colpo, Luis F. Torres, Sung-Min Cho, Aaron M. Gusdon, Louise D. McCullough, H. Alex Choi

**Affiliations:** ^1^Departent of Neurosurgery, McGovern Medical School, The University of Texas Health Science Center at Houston, Houston, TX, United States; ^2^Department of Neurology, McGovern Medical School, The University of Texas Health Science Center at Houston, Houston, TX, United States; ^3^Department of Neurology, Johns Hopkins University, Baltimore, MD, United States

**Keywords:** pain, COVID-19, long-haul, neurological symptoms, fatigue

## Abstract

COVID-19 is an ongoing pandemic with a devastating impact on public health. Acute neurological symptoms have been reported after a COVID-19 diagnosis, however, the long-term neurological symptoms including pain is not well established. Using a prospective registry of hospitalized COVID-19 patients, we assessed pain and neurological function (including functional, cognitive and psychiatric assessments) of several hospitalized patients at 3 months. Our main finding is that 60% of the patients report pain symptoms. 71% of the patients still experienced neurological symptoms at 3 months and the most common symptoms being fatigue (42%) and PTSD (25%). Cognitive symptoms were found in 12%. Our preliminary findings suggests the importance of investigating long-term outcomes and rationalizes the need for further studies investigating the neurologic outcomes and symptoms of pain after COVID-19.

## Introduction

To date over 75 million people have been infected with the *severe acute respiratory syndrome coronavirus 2* virus (SARS-CoV-2), which causes the coronavirus disease 2019 (COVID-19). While the vast majority will survive, many may be left with residual effects. One of the earliest studies reported acute neurological manifestations in hospitalized patients with COVID-19 that include encephalitis, acute myopathic quadriplegia, strokes and seizure (1). Some neurologic symptoms that these hospitalized patients experienced include headache, impaired consciousness, taste and smell impairment (2). Anecdotal reports of long-term neurologic symptoms are emerging as well (3). These reports have emphasized the importance of studying long-term neurologic outcomes, also referred as the “Long-Haul COVID” (4). Long-haul COVID (or post-COVID conditions) is defined by the Center for Disease Control as a wide range of new conditions or returning ones, or ongoing health problems that can persist for more than 4 weeks after the COVID-19 infection (5). Short- and long-term neurologic symptoms were reported during the SARS and MERS outbreaks (6) and it is increasingly evident that long-term symptoms will persist after COVID-19 as well (7). To characterize long-term neurologic outcomes after COVID-19 we followed a cohort of hospitalized patients and assessed 3 months outcomes.

## Methods

### Study Population and Patient Inclusion and Exclusion Criteria

This is a prospective study of COVID-19 patients admitted and hospitalized at the Memorial Herman Hospital System in Houston, Texas, USA. Inclusion criteria were laboratory-confirmed SARS-CoV-2 infection by real-time polymerase chain reaction, written informed consent from the patient or surrogate, and age ≥ 18 years. Exclusion criteria were inability to complete long-term follow-up, severe functional disabilities before hospital admission for COVID-19 [defined by pre-admission modified Rankin Score (mRS) >1] (8), history of pulmonary complications (including resection and transplant), pre-existing systemic diseases which would impact long term outcomes (including stroke, myocardial infarction, pulmonary disease requiring home oxygen, chronic renal failure necessitating hemodialysis and malignancy), documented neurologic and psychiatric disorders, prisoners and pregnant women. Patients were categorized as mild (nasal cannula with <5 liters of O_2_ and <5 days of hospitalization), moderate (nasal cannula with >5 liters of O_2_ or heat high flow cannula and > 5 days of hospitalization) and severe (on ventilator or expired). This study was approved by the “Institutional Review Board” (IRB No: HSC –MH-17-0452) at The University of Texas Health Science Center at Houston, Houston, Texas.

### Assessments

We assessed the functional, cognitive and psychiatric symptoms at 3 months after hospitalization. Pain, fatigue and sleepiness were evaluated using the Pain, Enjoyment of life and General activity (PEG) (9), the Fatigue Severity Scale (FSS) (10) and Epworth Sleepiness Scale (ESS) (11). Post-traumatic stress disorder was evaluated using the Primary Care PTSD Screen for DSM-5 (PC-PTSD-5) (12). Functional outcome was evaluated using the modified Rankin Score (mRS) (13). Cognitive status was evaluated using the brief neurocognitive screening test (BNST). Depression symptoms were evaluated using the Patient Health Questionnaire (PHQ-9) (14). Anxiety symptoms were assessed using the Generalized Anxiety Disorder (GAD-7). The battery of assessments, the range of the values, the cutoff and the interpretation are listed in Table 1. The primary rationale for the selection of the assessments include the ease of administering them over the phone and the limited time required to get the results. The entire battery of tests typically takes about 30–45 min to administer over the phone by a trained research personnel.

**Table 1:** Battery of assessments.

	**Range**	**Cutoff**	**Interpretation**
**Quality of life**			
Enjoyment of life and General activity (PEG)	0–10	n/a	Measure of pain and its interference in day-to-day activities. A continuous score that is typically tracked over time
Fatigue Severity Scale (FSS)	0–7	≥4 indicates fatigue	A 9-item questionnaire to quantify the degree of fatigue and its interference with day-to-day activities.
Epworth Sleepiness Scale (ESS)	0–24	≥11 indicates sleepiness	A 8-item tool to quantify the tendency to doze off.
PTSD Screen for DSM-5(PC-PTSD-5)	0–5	≥3 indicates risk for PTSD	A 5-item screen to identify individuals with probable PTSD
**Functional and cognitive outcomes**.			
Modified Rankin Score (mRS).	0–6	≥2 indicates continual neurological impairment	Measure of independence after a neurological disability or injury like stroke.
Brief Neurocognitive ScreeningTest (BNST)	0–12	≤ 8 indicates mild cognitive impairment	A 4-item questionnaire based off the MOCA that can be administered over the phone.
**Depression and anxiety:**			
Patient Health Questionnaire (PHQ-9)	0–20	≥10 indicates presence of depressive symptoms	A 9-item questionnaire to assess depressive symptoms
Generalized Anxiety Disorder (GAD-7) (15)	0–21	≥10 indicates presence of anxiety symptoms	A 7-item questionnaire to assess the symptoms of anxiety

### Statistical Analysis

Descriptive statistics were calculated for demographic variables in COVID-19 subjects. To describe differences in demographics, χ2-test, Fisher's exact test, student's *t*-test, and the Mann-Whitney U test were used where appropriate. The Mann-Whitney U test was used to test for significance across different groups A *p*-value of ≤ 0.05 was considered statistically significant (two-tailed). All statistical analyses were performed using open-source software packages in R (v3.1.3).

## Results

### Patient Demographics

One Hundred and forty Subjects Were Enrolled. 55 Were Lost to Follow-up and 27 Were Dead. 3 Month Outcomes Were Determined in 58 Subjects Using Telephone Questionnaires. The Average age of the Patients Were 49.2 ± 16 and 46% of the Subjects Were Female. 72% Were Hispanic-Reflective of the Large Hispanic Population at Houston. A Majority of the Patients Has co-Morbidities With 53% Being Obese, 38% With Diabetes and 46% With a History of Hypertension. The COVID-19 Symptoms of 44% of the patients were considered severe.

### Neurologic Symptoms

71% had continued neurologic symptoms highlighting the importance of considering the *long-haul COVID* phenomena. The most common symptom was pain (60%) followed by fatigue (41%) and PTSD symptoms (25%) (Table 2). There were no significant associations between PTSD and, ethnicity and race (p>0.05). People with long-term neurological symptoms were significantly older [mean, years (SD): 54 (16) vs. 41 (16); *p* = *0.01*]. The persistence of long-term symptoms was not associated with the severity of acute COVID-19 symptoms. Neither the maximum C-reactive protein levels [(137 (73) vs. 153 (92); *p* = *0.59*] nor the clinical severity [WHO ≤ 4 vs. WHO > 5, *p*=*0.58*] were associated with 3 month symptoms. In fact, we found that even subjects with mild course of hospitalization had a high incidence of symptoms, especially fatigue (58%).

**Table 2:** Demographics and assessments.

**Demographics**	***N* = 58**	
Age (mean, sd)	49.2 (16)	
Sex (Female, %)	27 (46)	
Ethnicity (Hispanic, %)	42 (16)	
**Past medical history**		
Obesity (*n*, %)	31 (53)	
Diabetes	22 (38)	
Hypertension	27 (46)	
Current Smoker	6 (10)	
COPD	2 (3.4)	
CCI (median, IQR)	2 (1–3)	
WHO Classification (Severe, %)	21 (44)	
**Outcomes**	***N*** **, %**	**Responses**
Any neurologic symptom	4 (71%)	58
Pain (PEG)	32 (60%)	53
Fatigue symptoms (FSS)	22 (41)	53
Post-traumatic stress disorder (PC-PTSD-5)	13 (25%)	52
Poor Functional outcome (mRS)	9 (16%)	56
Sleepiness (ESS)	7 (14%)	50
Cognitive Deficit (BNST)	6 (11.7%)	51
Depression Symptoms (PHQ-9)	8 (14%)	54
Anxiety (GAD-7)	6 (11.11%)	54

*The mRS is a 0–6 scale with a cutoff of ≥3 indicative of functional disability. BNST is a 0–12 scale with a cutoff of ≤ 8 indicative of cognitive symptoms. The PHQ-9 is a 0–20 with a cutoff of ≥10 indicative of depression symptoms. The GAD-7 is a 0–21 with a cutoff of ≥ 10 is indicative of general anxiety syndrome. PEG is a 0–10 point scale with no cutoff. FSS is a 0–7 point scale with a cutoff of ≥4 indicative of fatigue and ESS is a 0–24 point scale with a cutoff of ≥11 indicative of sleepiness symptoms. The PC-PTSD-5 is a 0–5 point scale with a cutoff of ≥3 indicative of PTSD)*.

### Pain Symptoms

**Figure 1:**
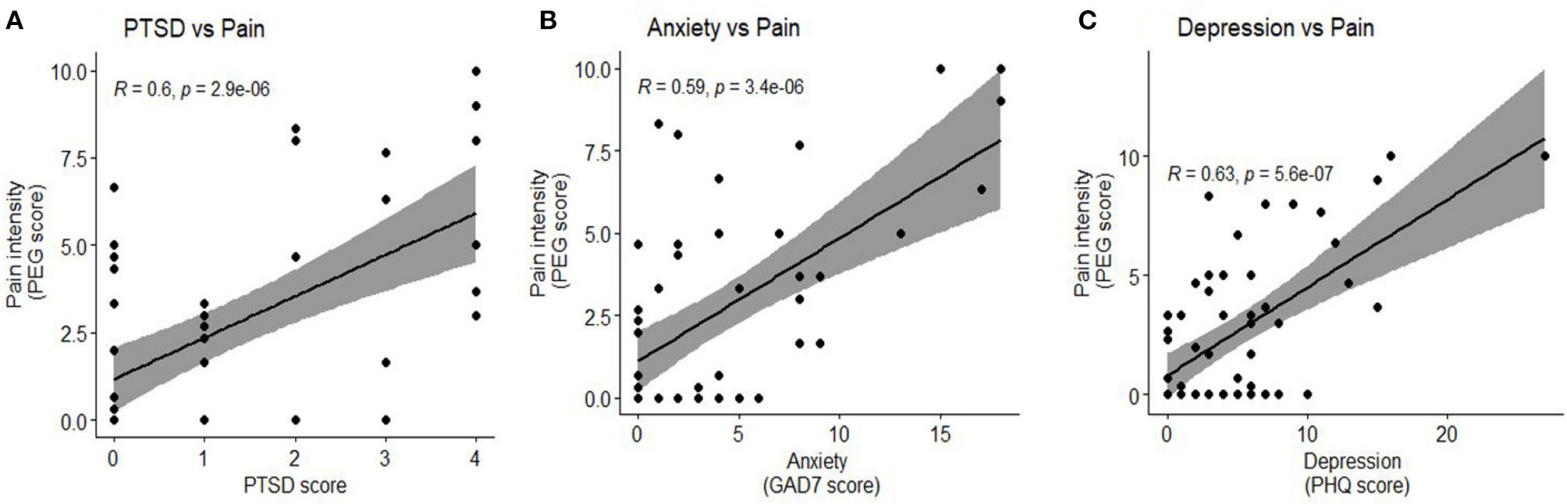
Association of Pain with PTSD, anxiety and depression. **(A)** Pain intensity had a significant positive correlation with PTSD score (*r* = 0.6, *p* < 0.01) **(B)** Pain intensity had a significant positive correlation with anxiety, quantified by the GAD7 score (*r* = 0.59, *p* < 0.01) **(C)** Pain intensity had a significant positive correlation with depression, quantified by the PHQ score (*r* = 0.63, *p* < 0.01).

In our cohort of hospitalized COVID-19 survivors, 60% of the subjects reported persistent pain symptoms. The most common location of pains were back, chest and head and 31% reported pain in two or more locations (Table 3). People who reported headache also reported the highest PEG score [median (IQR): 7.6(5–8.3)] compared to others (Table 3). Next, we compared the clinical characteristics and the assessments across subjects who did and did not report pain (Table 4). Interestingly, subjects who reported pain symptoms were significantly younger than those who did not report pain (mean ± SD: 45 ± 16 vs. 53 ± 11, p < 0.05). Women were also more likely to report pain symptoms-though this relationship was not statistically significant (*p* = 0.08). There were no differences in ethnicity and past medical history (including obesity, diabetes and smoking status) across the pain and no-pain subjects. Subjects who reported pain were also likely to experience other neurological and psychiatric symptoms at 3 months. Patients with pain were twice as likely to report symptoms of fatigue (53 vs. 23%, *p* < 0.05) and were seven times likely to be assessed with PTSD symptoms (37 vs. 4.7%, *p* < 0.01) than those who reported no pain. There was a strong and significant and positive correlation between pain and PTSD symptoms (*r* = 0.6, *p* < 0.01, Figure 1A). Anxiety and depression symptoms were positively and significantly correlated with pain symptoms (*r* = 0.59, *p* < 0.01 and *r* = 0.63, *p* < 0.01 respectively, Figures 1B,C). The pain subjects were also likely to have poor neurological outcomes and interestingly all patients who reported no pain had good neurological outcomes at 3 months. Subjects who reported pain were also more likely to report sleepiness, cognitive deficits, and, depression and anxiety symptoms, though these associations were not statistically significant.

**Table 3:** Pain location in 35 of the 58 subjects.

**Pain Location**	***N* (%)**	**Intensity [PEG, median(IQR)]**
Back	6 (17)	6.3 [3.6–8]
Chest	5 (14.2)	3 [1.6–5]
Headache	5 (14.2)	7.6 [5–8.3]
Other location	8 (22.8)	5 [3.5–8]
Two or more locations^*^	11 (31.4)	4.8 [3.5–8]

* *One patient reported whole body pain. IQR, Interquartile range*.

**Table 4:** Clinical variables and outcomes across pain and no-pain.

**Demographics**	**No-pain** **(*n* = 21)**	**Pain** **(*n* = 32)**	***p-*value**
Age (mean, sd)	53 (11)	45 (16)	**0.04**
Sex (Female, %)	6 (28)	18 (56)	0.08
Ethnicity (Hispanic, %)	18 (85)	22 (68)	0.2
**Past medical history**			
Obesity (*n*, %)	12 (57)	13	0.37
Diabetes	13 (62)	20	1
Hypertension	14 (67)	14	0.17
Current Smoker	18 (85)	29 (90)	1
CCI (median, IQR)	2 (1–3)	2 (1–3)	0.72
**Outcomes**			
Fatigue symptoms (FSS)	5 (23)	17 (53)	**0.04**
Post-traumatic stress disorder (PC-PTSD-5)	1 (4.7)	12 (37)	** <0.01**
Poor Functional outcome (mRS)	0 (0)	6 (19)	**0.07**
Sleepiness (ESS)	1 (4.7)	6 (19)	0.2
Cognitive Deficit (BNST)	2 (9)	4 (12)	1
Depression Symptoms (PHQ-9)	1 (4.7)	7 (22)	0.12
Anxiety (GAD-7)	0 (0)	5 (15.6)	0.14

*The bolded values indicate statistical significance*.

## Discussion

It is know that human coronaviruses (HCoVs) are neuro-invasive and neurotropic and have been shown to contribute to both short- and long-term neurological symptoms (6, 16, 17). Early studies have reported acute neurologic and psychiatric complications at COVID-19 hospitalization and these symptoms (18) include ischemic stroke, intracerebral hemorrhage, hallucinations, encephalopathy, anosmia and ageusia (19–24). Autopsy cases after COVID-19 (2) have shown brain injury after death. However, studies are underway to investigate the whether there are persistent long-term symptoms.

We undertook a study to investigate persistent symptoms at 3 months after COVID-19 hospitalization. Our main finding is that 60% of the patients experienced pain at 3 months after hospitalization. 71% of the patients still experienced neurological symptoms at 3 months and the most common symptoms being pain (60%), fatigue (41%) and PTSD (25%). The symptoms of pain were also associated with other symptoms like PTSD, anxiety and depression (Figure 1, Table 4) suggesting a relationship between these symptoms in recovering subjects. This relationship is consistent with reports in other conditions as well (25, 26) and also in survivors of the SARS pandemic of 2005 (27). Cognitive symptoms were found in 11.7% of the subjects. Our findings support the anecdotal reports of long-term symptoms even in those with mild acute pulmonary symptoms. The prevalence of cognitive symptoms (11.7%) were relatively lower than generalized symptoms. However, 11.7% of survivors represents a large number of people altogether, and gives credence to the reports of “brain fog” that survivors have experienced. Although symptoms of pain were frequent (60%), there is no standardized cut off for pain measurements so was not included in our analysis of combined neurologic symptoms. In those subjects who described pain, the PEG score was 4.42 ± 2.8 (mean ± SD), a score similar to ones reported in other diseases in which pain is prevalent after hospital discharge (28).

Interestingly, subjects who reported pain at 3 months were younger and tend to be female compared to those subjects who did not report pain. This demographic trend i.e., younger female and pain has been previously reported in fibromyalgia (29). Additionally, the subjects who reported pain in our cohort were also likely to be screened with psychiatric and neurologic comorbidities-a relationship that has been observed in fibromyalgia as well (29). Other studies in fibromyalgia have shown that women are at a greater risk at developing pain and that this sex dependent disparity could be due to factors including hormonal influences and gender expectations (30). Besides the incidence of pain being high in women compared to men, among those who reported pain, the degree of reported pain was also higher in women compared to men (4.63 ± 2.7 vs. 4.16 ± 2.94, *p* = 0.08, not shown in table). It is known that COVID-19 disproportionately affects men compared to women and some studies have shown that while the prevalence of infection is the same in men and women, men are more likely to be hospitalized and have a severe course of disease and death with a case fatality rate of 1.5–1.8 times that of women (31, 32). While gender-related behaviors such as smoking, drinking, the propensity to seek hospital care and presence of co-morbidities could affect the outcome of COVID-19, the presence of innate biological risk determinants could impact the outcomes as (33, 34). A number of reasons have been postulated for the differential response across sex to COVID-19. Differences in hormone levels and the immune response may underlie the differential effects by sex of infection (31). Sex differences are well-known in innate and adaptive immunity with resulting sex-specific responses to vaccines and infections (33). The fluctuation of sex steroid hormones that occurs over the life span also contributes to different disease susceptibility at different ages and to changes in immune profiles. In fact, the ACE2 receptor, a main SARS-CoV-2 (the virus that induces COVID-19) viral entry receptor, is X chromosome encoded, and is downregulated by estrogens (35). Higher levels of innate immune cytokines were associated with worse disease progression in women, but not in men. These early studies on sex differences in immunity to SARS-CoV-2 demonstrate that the immune landscape is different in males and females.

While hormonal differences and early immune response to COVID-19 between men and women can plausibly account for the acute disease severity and long-term outcomes, the underlying mechanisms of the higher incidence and degree of pain in women compared to men is unclear and warrants a detailed investigation.

### Limitations

The battery of questionnaires used in the study, while useful in screening patients with probable risk, are not conclusive in determining the status of the subject. Detailed and thorough in-person assessments are required to conclusively ascertain whether a clinical diagnosis of the condition is present. One aspect that governed the choice of the chosen assessments in the relative ease in which they can be administered–over the phone with relatively less training for the personnel who administer the assessments. Since COVID-19 is a relatively new disease, it not known *a priori* what symptoms that recovering subjects are experiencing. Therefore a broad range of assessments–pain, cognitive, functional, QoL and psychiatric–were administered. Additionally, these assessments take about 30–45 min to administer over the phone. We limited the assessment period to this time window (30–45 min) as we observed that some subjects were experienced “respondent fatigue” when the assessments took longer than that.

The findings of this study is limited to a cohort of hospitalized patient and results cannot be extrapolated to patients who experienced milder forms of COVID-19 that did not require hospitalization. Hospitalized Covid-19 patients, besides a higher degree of disease severity, are afflicted with a host of secondary complications including organ damage, invasive procedures and complications due to increased hospitalization. The disease pathophysiology is different from that of non-hospitalized patients. Despite the small sample size, this is one of the first study to prospectively evaluate long-term neurologic effects seen in COVID-19 patients. All the assessments were performed using phone questionnaires instead of in-person assessments. However, remote contactless assessments are necessary as the pandemic has changed the care paradigm (causing disruption in routine health care services) and many recovering patients are reluctant to participate in an in-person follow-up due to health limitations. The influence of age and co-morbidities were not analyzed due to the small sample size. Furthermore, this study does not include a suitable control cohort.

## Conclusion

Although studies have reported acute neurological symptoms after COVID-19, our study is one of the first to examine the persistence of neurologic symptoms and pain at 3 months after hospitalization. Studies examining pathophysiology and the time course of persistent symptoms after COVID-19 are needed. Our findings emphasize the importance of continued evaluation and focused rehabilitation for pain, functional, cognitive and neurobehavioral consequences in COVID-19 survivors.

## Data Availability Statement

The raw data supporting the conclusions of this article will be made available by the authors, without undue reservation.

## Ethics Statement

The studies involving human participants were reviewed and approved by the Institutional Review Board, The University of Texas Health Science Center at Houston. The patients/participants provided their written informed consent to participate in this study.

## Author Contributions

JS, LT, AG, LM, and HC were involved in the conception and design of the study. AB, SH, AP, AA, SJ, and GC were involved in the acquisition and analysis of data. JS, LT, AG, LM, S-MC, and HC were involved in the analysis of data. JS, S-MC, LT, AG, LM, and HC contributed substantially in drafting the manuscript. All authors contributed to the article and approved the submitted version.

## Conflict of Interest

The authors declare that the research was conducted in the absence of any commercial or financial relationships that could be construed as a potential conflict of interest.

## Publisher's Note

All claims expressed in this article are solely those of the authors and do not necessarily represent those of their affiliated organizations, or those of the publisher, the editors and the reviewers. Any product that may be evaluated in this article, or claim that may be made by its manufacturer, is not guaranteed or endorsed by the publisher.
